# Assessment of Oxidative Stress-Related Markers and Inflammatory Proteins in Serum and CSF Samples of Dogs with Different Types of Epilepsy

**DOI:** 10.3390/antiox15030282

**Published:** 2026-02-25

**Authors:** Rania D. Baka, Argyrios Ginoudis, Maria Botia, Juan Diego Garcia-Martinez, Ioannis Savvas, Dimitra Giota, Zoe Polizopoulou

**Affiliations:** 1 Companion Animal Clinic, School of Veterinary Medicine, Faculty of Health Sciences, Aristotle University of Thessaloniki, 54627 Thessaloniki, Greece; isavvas@gmail.com; 2 Diagnostic Laboratory, School of Veterinary Medicine, Faculty of Health Sciences, Aristotle University of Thessaloniki, 54627 Thessaloniki, Greece; ginoudisa@gmail.com (A.G.); dgiotavet@gmail.com (D.G.); poliz@vet.auth.gr (Z.P.); 3 Interdisciplinary Laboratory of Clinical Analyses (Interlab-UMU), Veterinary School, Campus of Excellence Mare Nostrum, University of Murcia, Campus Espinardo, 30100 Murcia, Spain; maria.botiag@um.es (M.B.); juandi@um.es (J.D.G.-M.)

**Keywords:** cerebrospinal fluid, c-reactive protein, cholinesterase, dogs, epilepsy, oxidative stress markers, oxytocin, serum

## Abstract

Background: Oxidative stress contributes to the development and progression of epilepsy and is connected with neuroinflammation during epileptic seizures. Cholinesterase has a modulatory role, and oxytocin has antiepileptic properties. The purpose of this study was to assess selective inflammatory (C-Reactive Protein, CRP) and oxidative stress markers [Paraoxonase-1 (PON1), cupric reducing antioxidant capacity (CUPRAC), ferric reducing antioxidant power (FRAP), cholinesterase, and oxytocin in serum and cerebrospinal fluid (CSF) samples of dogs with different types of epilepsy. Methods: There were four groups of dogs; A: healthy controls; B: idiopathic epilepsy receiving antiepileptic medication; C: idiopathic epilepsy without antiepileptic medication; and D: structural epilepsy. CRP, PON1, CUPRAC, and cholinesterase were evaluated in serum and PON1, CUPRAC, FRAP, cholinesterase and oxytocin were evaluated in CSF samples. Group differences were evaluated using the ANOVA test, followed by post hoc analyses or Kruskal–Wallis/Dunn’s test. Results: Fifty-one serum and 26 CSF samples were analyzed. CSF PON1 was significantly different in group D compared with groups A and C (*p* = 0.044 and *p* = 0.008, respectively). CSF cholinesterase was significantly different in group D compared with groups A, B and C (*p* = 0.003, *p* = 0.025, and *p* = 0.033, respectively). Conclusions: Structural epilepsy may influence PON1 and cholinesterase levels in CSF samples. Compared with CSF, serum was not the most suitable biological material to investigate oxidative stress and inflammatory markers.

## 1. Introduction

Epilepsy is a brain disease clinically manifested by epileptic seizures in both humans and animals [1,2]. The International League against Epilepsy (ILAE) has introduced a system to classify epileptic seizures according to their etiology in metabolic epileptic seizures, structural epilepsy, idiopathic epilepsy and genetic epilepsy [3]. Idiopathic epilepsy is most commonly diagnosed in young purebred or mixed-breed dogs and can be compared to human temporal lobe epilepsy or human idiopathic generalized epilepsy [4,5,6,7,8]. Structural epilepsy is mainly diagnosed in adult and aged dogs; in these age groups, inflammatory encephalopathy is being diagnosed most frequently in small-breed dogs and extra-axial neoplasia in large-breed dogs [9,10]. Diagnosis of the different types of epilepsy is based on advanced diagnostic imaging and/or cerebrospinal fluid (CSF) analysis [11]. The application of magnetic resonance imaging (MRI) in veterinary medicine has revealed many brain structural and functional abnormalities; however, it cannot identify and explain the molecular basis of the spontaneous abnormal electrical discharge of neurons that causes epileptic seizures in idiopathic epilepsy [9,10,12,13,14,15,16]. Electroencephalography (EEG), a routine diagnostic test in human patients with epilepsy, can identify the locus of the abnormal electrical discharge in the specific lobe of the brain; however, its use is limited in veterinary medicine [17].

Regarding epileptogenesis, while significant progress has been recorded in recent years, many aspects of the underlying mechanisms remain unclear [18,19]. Oxidative stress occurs when there is an imbalance between the reactive oxygen species (ROS) or reactive nitrogen species (RNS) and the brain’s antioxidant defenses [20,21]. In the brain, which has high oxygen consumption and relatively low antioxidant capacity, oxidative stress is especially damaging. Therefore, oxidative stress has a significant role in the development and progression of epilepsy, particularly in the process of epileptogenesis and seizure-induced brain damage [22]. Oxidative stress has been assessed with specific biomarkers in many studies in blood or brain tissue from patients with epilepsy or from animal models exhibiting *status epilepticus* [22,23,24,25,26,27,28]. However, the bibliography is limited regarding the assessment of oxidative stress markers in cerebrospinal fluid (CSF), which could be the ideal material to study through its direct contact with the brain [29]. Oxidative stress is strongly connected with neuroinflammation during epileptic seizures and epilepsy. It forms a vicious cycle, where each process amplifies the other and contributes to neuronal damage, epileptogenesis and seizure recurrence [22,30,31]. Many studies have evaluated inflammatory markers and particularly C-reactive protein (CRP) in blood and CSF samples of humans and animals with neurological disorders including epilepsy [32,33,34,35,36,37,38,39,40,41,42,43,44,45,46]. Blood CRP is found elevated in patients with epilepsy compared with controls, and antiepileptic medication can reduce CSF and blood CRP levels [24,32,33,35,36,38,40,41,42,43]. Elevated CRP levels are found in CSF of dogs affected with distemper and in serum samples of dogs with *status epilepticus* due to idiopathic epilepsy [44,45,46].

Cholinesterase does not play a primary active role in epilepsy; it can have an indirect/modulatory role through its impact on acetylcholine (AChE) levels, which affect neuronal excitability. Therefore, if cholinesterase activity is inhibited, AChE accumulates, leading to neuronal overexcitation, triggering epileptic seizures or *status epilepticus* [47,48]. The bibliography is limited regarding the assessment of cholinesterase in human patients with epilepsy, probably because of its indirect association with epilepsy. There is a paper indicating elevated cholinesterase activity in patients with epilepsy and decreased cholinesterase levels in the blood and CSF of patients with epilepsy after surgical treatment [47].

Oxytocin has been evaluated for its antiepileptic properties, mostly in experimental studies of patients with epilepsy, as well as in patients with other mental co-morbidities [49,50,51,52,53]. In veterinary medicine, research regarding oxytocin has been performed in mice and no published data in dogs have been identified [51,53]. Canine epilepsy shares many clinical and pathophysiological similarities with human epilepsy, therefore a canine model should be considered ideal to study the therapeutic potential of oxytocin in canine epileptic patients.

The current study aimed to assess oxidative stress and inflammatory markers in serum and cerebrospinal fluid (CSF) samples of dogs naturally affected by idiopathic epilepsy. In addition, oxytocin was measured and a new assay for its quantification in CSF was validated.

## 2. Materials and Methods

### 2.1. Ethical Approval

This prospective, cross-sectional study involved dogs admitted to the School of Veterinary Medicine, Faculty of Health Sciences, Aristotle University of Thessaloniki, Greece from March until November 2018. The study population included four groups. European legislation on animal handling and experiments was followed (86/609/EU). The study was approved by the ethical committee (Prot. No. 567/13/03/2018). The owners of epileptic dogs were fully informed of the proposed diagnostic protocol (clinicopathological and diagnostic imaging testing) and gave their written informed consent prior to participation in this study.

### 2.2. Study Population

Group A included clinically healthy dogs (control group) without a history of epileptic seizures or any other systemic disease. Recruitment was conducted through a stray animal spaying/neutering program after written consent was obtained. Blood collection and brain imaging were carried out before the spaying/neutering procedure.

The remaining three groups (Groups B, C, and D) comprised dogs admitted with a history of recurrent epileptic seizures or as emergency cases presenting with *status epilepticus*. The allocation of dogs into these groups was performed following the completion of a thorough diagnostic evaluation. In cases where the diagnostic work-up did not reveal any structural abnormalities, the age at seizure onset was compatible (>6 months and <5 years), and if a history of recurrent epileptic seizures was present, a diagnosis of idiopathic epilepsy was considered highly suggestive [54]. Group B included dogs diagnosed with idiopathic epilepsy that were receiving antiepileptic medication at the time of admission, whereas Group C consisted of dogs with idiopathic epilepsy that were not receiving antiepileptic medication upon admission. Group D comprised dogs diagnosed with structural epilepsy. The age at seizure onset ranged from 6 months to 5 years for dogs in Groups B and C, while no age restriction was applied for dogs in Group D. Prior administration of antiepileptic medication (AEM) was not considered an exclusion criterion. Both the type of antiepileptic medication and the duration of treatment were recorded. Some dogs in Group D were also receiving AEM at the time of admission. Given that both the initiation and duration of AEM were considered relevant factors, treatment duration was incorporated into the inclusion criteria. Accordingly, dogs receiving AEM at admission were included in this study only if the medication had been administered at an appropriate dosage and for a sufficient duration to ensure the attainment of therapeutic serum concentrations. For the antiepileptic medications used in the study population—phenobarbital (PB), levetiracetam (LEV), and bromide (Br)—the minimum treatment duration required was at least one month for PB and LEV, and at least three months for bromide [55,56]. Serum drug concentrations were monitored in dogs belonging to Groups B and D to evaluate therapeutic efficacy. Drug concentration analyses were performed by an external collaborating laboratory (IDEXX Laboratories, Kornwestheim, Germany).

Epidemiological data, as well as the age at seizure onset, seizure frequency, and seizure type, were systematically recorded (Supplementary Material, Table S1). For dogs receiving antiepileptic medication (AEM), additional data regarding therapeutic response, seizure frequency, and seizure type were also documented. Dogs weighing less than 2 kg, as well as dogs presenting with reactive seizures—defined as seizures secondary to systemic metabolic disturbances or exogenous toxic disorders identified either during history taking or clinicopathological evaluation—were excluded from this study. Additional exclusion criteria included an acute or previous history of head trauma, congenital disorders (e.g., hydrocephalus), and the presence of any other concurrent disease identified during the diagnostic work-up. A comprehensive medical history was obtained for each dog, including age at seizure onset, seizure frequency, type and duration of seizures, initiation of antiepileptic medication, previous laboratory evaluations, and prior brain diagnostic imaging. This information was supplemented by visual documentation of seizure episodes, provided by the owners in the form of video recordings, to differentiate epileptic seizures from other paroxysmal events that may mimic epileptic seizure activity.

Clinicopathological evaluation comprised complete blood count (CBC) analysis, serum biochemistry profiling, and urinalysis. Complete blood counts and serum biochemistry analyses were performed using the ADVIA 120 Hematology System (Bayer Diagnostics, Dublin, Ireland) and the Vitalab Flexor E analyzer (Spankeren, The Netherlands), respectively.

Diagnostic imaging evaluation comprised thoracic radiological and abdominal ultrasonographic examination. Dogs in which any concurrent systemic disease was identified during the diagnostic workup were excluded from this study. Brain imaging was performed using computed tomography (CT) (Optima 16-slice, GE Healthcare, Wuppertal, Germany) and/or magnetic resonance imaging (MRI) (SignaHDx 1.5 T, GE, Waukesha, WI, USA) under general anesthesia, with induction using propofol and maintenance using isoflurane.

### 2.3. Sampling

#### 2.3.1. Blood Sampling

Blood samples were collected from either the cephalic or the jugular vein and stored in serum separator tubes (Eurotubo, Deltalab, 0819, Rubi, Spain) before separation. After centrifugating (3000 × 8 min), serum samples (1 mL for each dog) were separated in aliquots and stored in Eppendorf vials (Hamburg, Germany), frozen at −80 °C for forthcoming analysis. Frozen samples were shipped for analysis as a single batch using special courier services and transport in containers with card ice.

#### 2.3.2. Cerebrospinal Fluid (CSF)

Cerebrospinal fluid (CSF) samples were collected via cisternal tap under general anesthesia and after confirmation from computed tomography (CT) or/and MRI brain imaging for the safety of the procedure. The collected amount of CSF was 1 mL/5 kg of body weight. CSF samples with iatrogenic blood contamination were excluded from this study. CSF analysis was performed within 30 min after collection and included total cell counts, measurements of total protein, and cytological examination. The cytological examination of CSF was performed in stained slides obtained using a cytocentrifuge (Aerospray Pro slide stainer/cytocentrifuge ELI Tech Group WESCOR, Logan, UT, USA) and the cell counts were performed microscopically using a hemocytometer (BLAUBRAND Neubauer improved, BRAND, Wertheim, Germany). CSF total proteins were measured in an automated biochemistry analyzer (FLEXOR Vitalab, Vital Scientific B.V., Spankeren, The Netherlands) using the pyrogallol red method (Dia Sys Diagnostic Systems, Grabels, France). The remaining CSF samples were centrifuged to remove cells and the supernatants were frozen at −80 °C for forthcoming analysis. Frozen samples were shipped for analysis as a single batch using special courier services and transport in containers with dry ice.

### 2.4. Sample Analysis

#### 2.4.1. Serum Sample Analysis

Paraoxonase 1 (PON1), cupric reducing antioxidant capacity (CUPRAC), cholinesterase and C-reactive protein (CRP) were assessed in serum samples in all 4 groups of dogs.

#### 2.4.2. CSF Sample Analysis

Paraoxonase 1 (PON1), CUPRAC, ferric reducing antioxidant power (FRAP), cholinesterase, and oxytocin were assessed in CSF samples. The limited volume of CSF collection was not sufficient for all five marker measurements; therefore, some data are missing.

#### 2.4.3. Methods

Serum and CSF Paraoxonase 1 (PON1) activity assays were assessed based on the hydrolytic activity of the enzyme in 4-nitrophenyl acetate substrate as previously described [57].

CUPRAC is a laboratory method that evaluates the reduction in cupric ions (Cu^+2^) to cuprous ions (Cu^+^) by antioxidant agents in the serum and CSF samples using a validated automated assay [58].

FRAP assay in CSF assessed the reduction of ferric-tripyridyltriazine (Fe^3+^-TPTZ) to the ferrous (Fe^2+^) following previously described methods [59,60].

The activity of cholinesterase was measured in serum and CSF samples using butyrylthiocholine as previously described [61].

CRP was measured with an immunoturbidimetric assay previously validated in dogs [62].

All the previous assays showed inter- and intra-assay imprecision values lower than 15 and linearity after serial sample dilution.

For oxytocin measurement, a direct competition assay based on AlphaLISA (PerkinElmer, Waltham, MA, USA) technology, in which acceptor beads are coated to a monoclonal anti-oxytocin antibody, was used. The monoclonal antibody used for assay development is previously described in a previous report about oxytocin measurement in pigs [63].

For analytical validation of the assay, imprecision was calculated as inter- and intra-assay variations and expressed as coefficients of variation (CVs). Five replicates of two samples with different concentrations (2443.68 and 485.31 pg/mL) were analyzed at the same time to determine the intra-assay precision of the method. Five aliquots of each sample were stored in plastic vials at −80 °C. These aliquots were measured in duplicate five times over five different days using freshly prepared calibration curves for inter-assay precision.

The accuracy was evaluated by an assessment of linearity under dilution and recovery experiments. For the linearity evaluation, two samples (2443.68 and 485.31 pg/mL) were serially diluted from 1:2 to 1:256) with AlphaLISA universal buffer.

The detection limit (LD) and lower limit of quantification (LLQ) were obtained to evaluate the sensitivity of the method. The LD was calculated as the mean of 10 replicate measurements of the assay buffer plus three standard deviations. For the LLQ, a serial dilution (from 1:2 to 1:256) of the cerebrospinal fluid sample (384.66 pg/mL) was performed, analyzing 5 replicates of each dilution in the same run. The CV was calculated for each dilution, establishing the LLQ as the lowest dilution that could be measured with <20% imprecision.

### 2.5. Statistical Analysis

#### 2.5.1. Serum Samples

Descriptive statistics were produced using Jasp 0.19.3. An ANOVA test was used to determine whether there was a significant difference for PON1, CUPRAC and cholinesterase among the 4 groups of dogs in serum samples. Post hoc comparisons were performed in parameters among the four groups. Dunn’s test, which followed the Kruskal–Wallis test, was also used to assess the significance of serum CRP.

#### 2.5.2. CSF Samples

Descriptive statistics were produced using Jasp 0.19.3. An ANOVA test was used to determine whether there was a significant difference of FRAP and CUPRAC among the dogs in CSF samples. Post hoc comparisons were performed in parameters between the groups. Dunn’s test, which followed the Kruskal–Wallis test, was also used to assess the significance of PON1, cholinesterase and oxytocin.

## 3. Results

### 3.1. Serum Samples

In total, 51 serum samples were analyzed for oxidative stress and inflammatory markers. Forty-three serum samples were collected from epileptic dogs: 15 serum samples from Group B, 11 serum samples from Group C, and 17 samples from Group D dogs. The remaining eight serum samples were collected from healthy controls (Group A).

#### 3.1.1. Serum Oxidative Stress Markers

**Figure 1:**
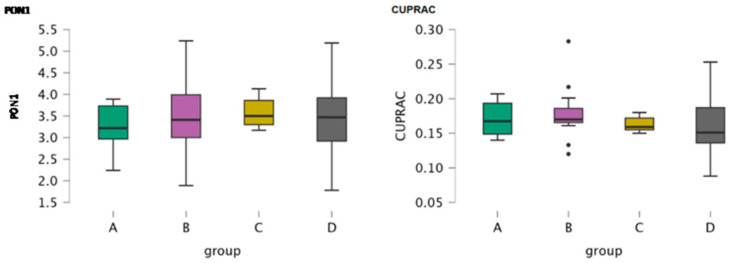
Boxplots of the concentration of the oxidative stress parameters (Paraoxonase 1, PON1 and cupric reducing antioxidant capacity, CUPRAC) in serum samples of the four study groups (A: clinically healthy dogs, B: dogs with idiopathic epilepsy that were receiving antiepileptic medication at the time of admission, C: dogs with idiopathic epilepsy that were not receiving antiepileptic medication upon admission, D: dogs with structural epilepsy). Boxplot of each serum parameter (PON1, CUPRAC) depicts the maximum, the first quartile, the median, the third quartile and the minimum values of the parameter. Black points refer to outliers.

Paraoxonase 1 (PON1) and cupric reducing antioxidant capacity (CUPRAC) were assessed in serum samples of the four groups of dogs as markers of oxidative stress. Table 1 includes the mean, minimum and maximum values of PON1 and CUPRAC in the four groups. Boxplots depict the activity of PON1 and the concentration of CUPRAC in the four groups (Figure 1). An ANOVA test did not reveal any significance for PON1 and CUPRAC among the four groups of dogs (*p* = 0.719 and *p* = 0.602, respectively). Post hoc comparisons performed between the groups did not reveal any significance either for PON1 or for CUPRAC (Table 2).

#### 3.1.2. Cholinesterase

**Figure 2:**
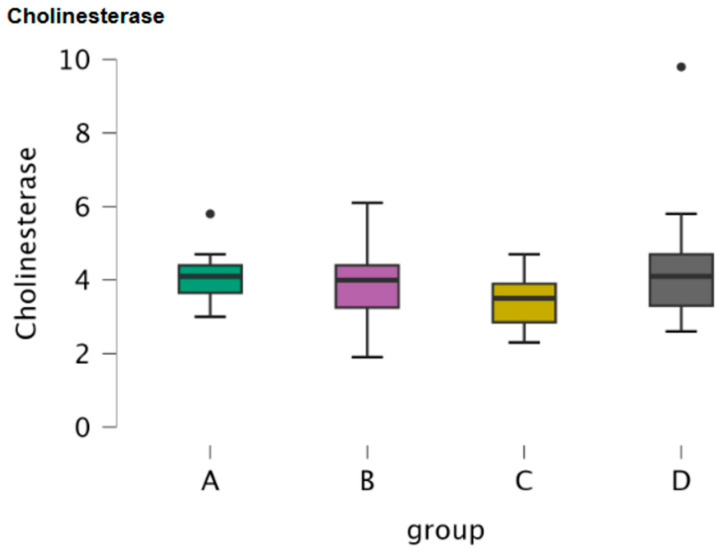
Boxplot of the concentration of cholinesterase in serum samples of the four study groups (A: clinically healthy dogs, B: dogs with idiopathic epilepsy that were receiving antiepileptic medication at the time of admission, C: dogs with idiopathic epilepsy that were not receiving antiepileptic medication upon admission, D: dogs with structural epilepsy). Boxplot of serum cholinesterase depicts the maximum, the first quartile, the median, the third quartile and the minimum values of the parameter. Black points refer to outliers.

Cholinesterase was assessed in serum samples of the four groups of dogs. Table 1 includes the descriptive statistics of cholinesterase. Boxplots illustrated the concentration of cholinesterase in the four groups of dogs (Figure 2). An ANOVA test did not reveal any significance of cholinesterase among the four groups of dogs (*p* = 0.321). Post hoc comparisons between the groups did not reveal any significance for cholinesterase (Table 2).

#### 3.1.3. C-Reactive Protein (CRP)

**Figure 3:**
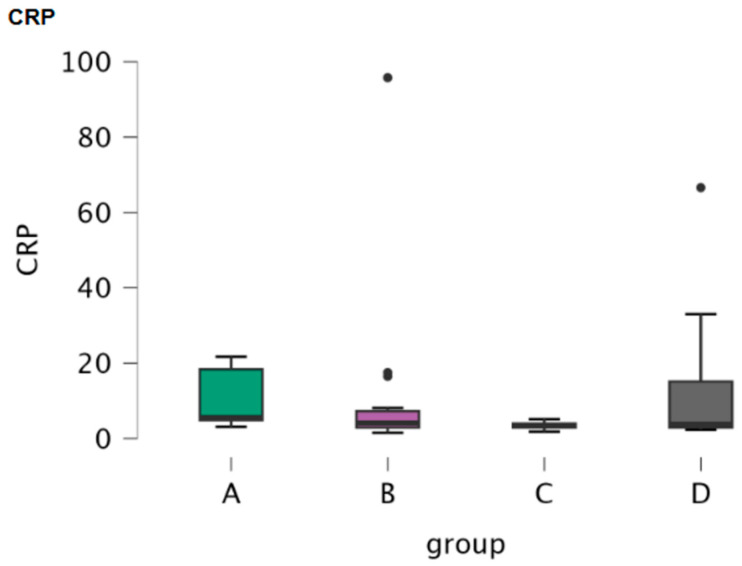
Boxplot of the concentration of CRP in serum samples of the four study groups (A: clinically healthy dogs, B: dogs with idiopathic epilepsy that were receiving antiepileptic medication at the time of admission, C: dogs with idiopathic epilepsy that were not receiving antiepileptic medication upon admission, D: dogs with structural epilepsy). Boxplot of serum CRP depicts the maximum, the first quartile, the median, the third quartile and the minimum values of the parameter. Black points refer to outliers.

C-reactive protein (CRP) was assessed in serum samples of the four groups of dogs. Table 1 includes the descriptive statistics of CRP. Boxplots illustrate the concentration of CRP in the four groups of dogs (Figure 3). Kruskal–Wallis and Dunn’s post hoc comparisons did not reveal any significance (Table 3 and Table 4).

### 3.2. Cerebrospinal Fluid (CSF) Samples

In total, 26 cerebrospinal fluid (CSF) samples were analyzed for oxidative stress and inflammatory markers. There was an inadequate CSF sample volume for all measurement assessments in some cases. Therefore, in the control group of dogs (Group A), PON1, FRAP, cholinesterase, CUPRAC and oxytocin were assessed in five samples. In idiopathic epilepsy dogs undergoing antiepileptic medication (Group B), PON1 and cholinesterase were assessed in four samples, and FRAP, CUPRAC and oxytocin in six samples. In idiopathic epilepsy dogs that did not receive any antiepileptic medication (Group C), PON1 was assessed in five samples, FRAP and cholinesterase in six samples, and CUPRAC and oxytocin in seven samples. In structural epilepsy cases (Group D), PON1 and cholinesterase were assessed in seven samples, and FRAP, CUPRAC and oxytocin in eight samples (Table 5).

#### 3.2.1. CSF Oxidative Stress Markers

**Figure 4:**
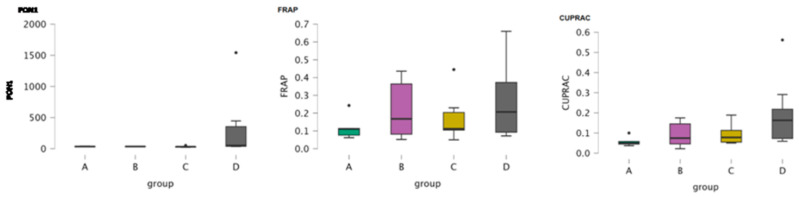
Boxplots of the concentration of oxidative stress markers (PON1, FRAP, CUPRAC) in CSF samples of the four study groups (A; clinically healthy dogs, B: dogs with idiopathic epilepsy that were receiving antiepileptic medication at the time of admission, C: dogs with idiopathic epilepsy that were not receiving antiepileptic medication upon admission, D: dogs with structural epilepsy). Boxplot of CSF PON1, FRAP, and CUPRAC depicts the maximum, the first quartile, the median, the third quartile and the minimum values of the parameters. Black points refer to outliers.

CSF oxidative stress markers’ (PON1, FRAP and CUPRAC) mean, minimum and maximum values are included in Table 5. Boxplots depict PON1 activity and FRAP and CUPRAC concentrations in the four groups (Figure 4). An ANOVA test did not reveal any significance of FRAP or CUPRAC among the four groups of dogs (*p* = 0.469 and *p* = 0.095, respectively). Post hoc comparisons performed between the groups did not reveal any significance for any of the two oxidative stress parameters (FRAP, CUPRAC) (Table 6). A Kruskal–Wallis test revealed a significant difference in PON1 between groups (*p* = 0.037) (Table 7) and Dunn’s test for PON1 that followed indicated significant differences between Groups A and D and between Groups C and D (*p* = 0.044 and *p* =0.008, respectively) (Table 8).

#### 3.2.2. Cholinesterase

**Figure 5:**
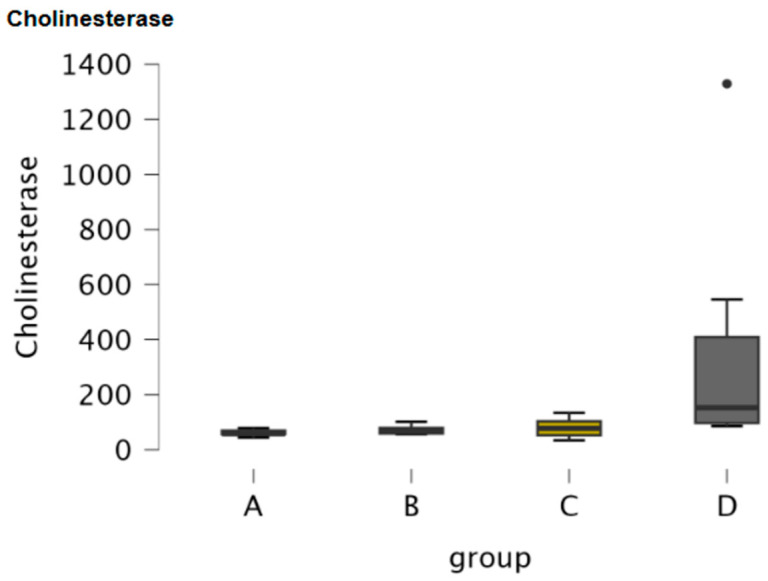
Boxplot of the concentration of cholinesterase in CSF samples of the four study groups (A: clinically healthy dogs, B: dogs with idiopathic epilepsy that were receiving antiepileptic medication at the time of admission, C: dogs with idiopathic epilepsy that were not receiving antiepileptic medication upon admission, D: dogs with structural epilepsy). Boxplot of CSF cholinesterase depicts the maximum, the first quartile, the median, the third quartile and the minimum values of the parameter. Black points refer to outliers.

Cholinesterase was assessed in CSF samples of the four groups of dogs. Table 5 includes the descriptive statistics of cholinesterase. Boxplots illustrate the concentration of cholinesterase in the four groups of dogs (Figure 5). Kruskal–Wallis revealed a significance of cholinesterase among the groups (*p* = 0.013) and Dunn’s post- hoc comparisons revealed significance between Groups A and D, Groups B and D and between Groups C and D (*p* = 0.003, *p* = 0.025, and *p* = 0.033, respectively) (Table 7 and Table 8, respectively).

#### 3.2.3. Oxytocin

The oxytocin assay demonstrated intra- and inter- assay CVs of 1.41–2.31% and 3.19–4.60%, respectively. The dilution of CSF samples resulted in linear regression equations, with a correlation coefficient of 0.99. The assay LD and LLQ were 2.14 and 39.27 pg/mL, respectively.

**Figure 6:**
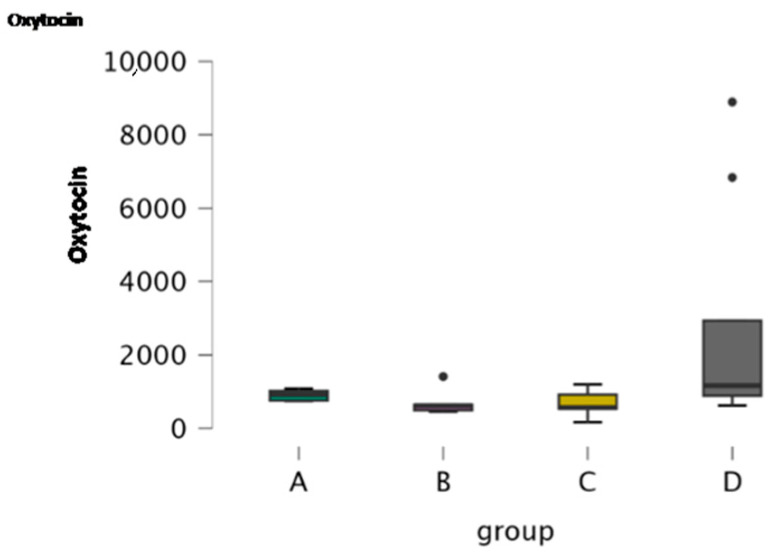
Boxplot of the concentration of oxytocin in CSF samples of the four study groups (A: clinically healthy dogs, B: dogs with idiopathic epilepsy that were receiving antiepileptic medication at the time of admission, C: dogs with idiopathic epilepsy that were not receiving antiepileptic medication upon admission, D: dogs with structural epilepsy). Boxplot of CSF oxytocin depicts the maximum, the first quartile, the median, the third quartile and the minimum values of the parameter. Black points refer to outliers.

Oxytocin was assessed in 26 CSF samples. Table 5 includes the descriptive statistics of oxytocin. The boxplot depicts the concentration of oxytocin in the four groups (Figure 6). Kruskal–Wallis revealed the significance of oxytocin among groups (*p* = 0.046) and Dunn’s post hoc comparisons revealed significance between Groups B and D and between Groups C and D (Table 7 and Table 8, respectively).

## 4. Discussion

Epilepsy is a complex disease entity that involves inflammatory and oxidative stress processes in addition to abnormal electrical activity [31]. In the current study, inflammatory markers (CRP), oxidative stress markers (PON1, CUPRAC, FRAP), cholinesterase and oxytocin were assessed in serum and CSF samples of epileptic dogs with different types of epilepsy.

Serum CRP can be temporarily increased in patients exhibiting generalized tonic–clonic seizures, *status epilepticus*, or prolonged seizures. This increase is modest unless there is another underlying condition [33,38]. Most patients with epilepsy have normal CRP, especially between seizures [38]. In the current study, CRP was assessed in serum samples of epileptic dogs. Median values were 5.55 μg/mL for Group A (control group), 4.1 μg/mL for Group B, 3.4 μg/mL for Group C, and 3.8 μg/mL for Group D. None of the median values exceeded the reference range for CRP in serum samples (<10 μg/mL). All comparisons among the four groups did not reveal any significant differences. Multiple human studies have indicated increased serum CRP values in patients with epilepsy compared with controls [41,42]. In particular, despite the increased serum CRP concentration in refractory epilepsy cases, CRP values were decreased when patients received antiepileptic medication but still remained increased compared with controls [33,35,37]. Levetiracetam antiepileptic treatment decreased serum CRP concentration compared with other antiepileptics [40,43]. In an experimental rat model assessing CRP at different time points after electrically induced *status epilepticus*, there were no concentration changes identified [34]. In contrast to this study, other studies involving epileptic dogs indicated increased serum CRP levels in dogs diagnosed with structural epilepsy compared with idiopathic epilepsy dogs and in dogs exhibiting *status epilepticus* [44,45]. In the current study, there was no significant difference in CRP levels among the three groups of epileptic dogs compared with controls. The time elapsing from the last seizure till serum sampling and the different antiepileptic medications administered (Group B and Group D dogs) could have influenced the results. In particular, concerning the time interval between the last seizure and serum sampling, it was not standardized for the study population; therefore, sampling was performed regardless of the time the last epileptic seizure occurred. Furthermore, no inflammatory encephalopathy cases were included in the structural epilepsy Group D. In a previously published study, including dogs diagnosed with distemper encephalitis, serum CRP levels were elevated compared with controls [46]. The results of the current study support evidence from human patients; CRP had been within reference ranges in patients with epilepsy suffering from tonic–clonic epileptic seizures [38]. Results from the current study indicate that CRP is not a reliable inflammatory marker for either idiopathic or structural epilepsy in dogs.

Oxidative stress has been associated with epilepsy in both human and canine patients [24,25,27,46]. Although there are multiple studies assessing oxidative stress in human neurological diseases, including epilepsy, the bibliography is limited in canine epilepsy [26,28,64,65]. In the current study, selective oxidative stress markers were evaluated in both serum (PON1 and CUPRAC) and CSF (PON1, CUPRAC, FRAP) samples of three groups of dogs diagnosed with different types of epilepsy and a control group (Group A). Paraoxonase 1 (PON1) has an important anti-inflammatory and antioxidant role; it protects lipids and lipoproteins from oxidative damage by preventing lipid peroxidation in cell membranes and lipoproteins [66,67]. In general, PON1 concentration was decreased in oxidative stress [66,67]. The overall assessment of median values of PON1 of the current study indicated that serum concentrations were much lower compared with CSF concentrations. To the authors’ knowledge, there is no available literature indicating the reference range of PON1 in serum or CSF in dogs with epilepsy. In the study of [65], where antioxidant markers, including PON1, in dogs with idiopathic epilepsy were assessed, it was concluded that serum PON1 was lower compared with healthy controls, but no reference ranges were provided. Contrary to the results of comparisons of the serum PON1 values among the four study groups, there was a statistically significant difference in CSF PON1 when healthy controls (Group A) and dogs with idiopathic epilepsy that did not receive antiepileptic medication (Group C) were compared with structural epilepsy (Group D). A possible explanation for this finding could be the severity of brain damage in Group D cases (structural epilepsy) and the demand for further antioxidant protection of the nervous tissue from further damage. Since PON1 cannot cross the blood–brain barrier (BBB), even if it is impaired [68], the results of the current study are an important finding that requires further investigation. The same research group mentioned that, despite the fact that there is no documented gene expression in mouse or human brain tissue, a hypothesis of transport of PON1 via “discoidal HDL” with unspecified mechanisms could not be excluded [68]. The BBB not only limits the passive diffusion of these markers but also influences the dynamics of redox homeostasis in the brain, often resulting in systemic markers that do not reflect the actual oxidative condition within the CNS [69]. There were additional studies of PON1 identification in CSF samples of patients suffering from neurodegenerative diseases and they speculate that CSF PON1 originated from the periphery [70,71]. Therefore, CSF PON1 identification, origin and mechanism of action in epilepsy need further investigation.

CUPRAC measurement is a reliable method for assessing the antioxidant capacity of a sample by reducing Cu^2+^to Cu^1+^ [58]. Therefore, decreased CUPRAC values may indicate reduced antioxidant defense in multiple diseases [58]. Limited data are available regarding the assessment of CUPRAC in human and canine epilepsy. Overall assessment of median CUPRAC values between the two different sample types (serum and CSF) indicates a tendency for higher CUPRAC values in serum compared with CSF (except for Group D). There was no significance identified in either serum or CSF CUPRAC among the four groups. To the authors’ knowledge, there are no other previously published papers assessing CUPRAC in patients with epilepsy.

FRAP (ferric reducing ability) is a method that assesses the antioxidant capacity of a sample by reducing ferric ion (Fe^3+^) to ferrous ion (Fe^2+^) [59]. In the current study, FRAP was evaluated in CSF. Statistical analysis did not reveal any significance of FRAP among the four groups. Previous studies reported increased serum and CSF FRAP values in canine patients with distemper encephalitis and decreased values in human patients diagnosed with Fabry disease [46,64]. Since the published literature is limited and involves different species (human vs. canine) and/or different disease entities, secure conclusions could not be extrapolated regarding FRAP in canine epilepsy.

Cholinesterase activity (acetylcholinesterase and butyrylcholinesterase) is correlated with epilepsy through cholinergic neurotransmission, which is closely linked to neuronal excitability and seizure activity [47,65]. In this study cholinesterase was assessed in both serum and CSF samples of epileptic dogs and healthy controls. Serum cholinesterase activity was not significant among the four study groups. On the contrary, CSF cholinesterase activity was significant when Group D dogs (structural epilepsy) were compared with the other two groups of idiopathic epilepsy (Groups B and C) and the control group (Group A). CSF cholinesterase activity is altered (increased) probably through a localized release in the brain, as a compensatory mechanism [72]. In this study, both serum and CSF median cholinesterase values are increased, but the increase in CSF is more prominent. Interestingly, an increase was also recorded in Group A (control group). A possible explanation could be that stress may be responsible since these dogs were thoroughly investigated and no abnormalities were identified during routine physical examination or clinicopathological testing. The bibliography supports the influence of acute stress episode on cholinesterase by increasing its activity in the brain and peripheral nervous system [73].

In this report an AlphaLISA assay for the measurement of oxytocin in CSF of dogs was analytically validated given the adequate values of precision and accuracy, indicating that this assay can be applied for oxytocin CSF quantification. In humans and rats, exogenous oxytocin administration (intranasally, intra-hippocampal microinjection) may reduce seizure severity and frequency on a long-term basis [49,50,51,52,53]. In this study, CSF endogenous oxytocin levels were evaluated in the four groups of dogs. There was a statistically significant increase in CSF oxytocin between Group D dogs compared with the other two groups of idiopathic epilepsy dogs (Groups B and C). This increase in Group D could be due to the presence of more severe brain lesions when structural epilepsy is suspected and could increase to compensate for the damage since it produces neuroprotection [53]. However, the small sample size of Group D dogs (eight dogs) necessitates further investigation in a larger animal population.

The limitations of the current study include missing data. Notably, the CSF sample size was modest (26 samples), and some biomarkers were unavailable in certain groups because of limited CSF volume, resulting in missing data. This reduces statistical power—particularly for subgroup comparisons—and constrains generalizability. Therefore, these results require confirmation in larger, adequately powered cohorts with more complete CSF profiling and independent validation. In addition, some limitations are related to the quantification and analytical interpretation of oxidative stress and inflammatory biomarkers. Single time-point measurements may not fully capture the dynamic fluctuations associated with seizure activity. Importantly, due to the clinical nature of this study and the inclusion of naturally occurring epilepsy cases, a strict control of sampling in relation to seizure timing was not feasible, reflecting real-world clinical conditions. Despite these inherent constrains, the study design was strengthened by standardized sample processing, batch analysis of samples, and use of validated analytical assays, supporting the internal consistency and reliability of the findings. The heterogeneity of the study population with respect to antiepileptic medications, which constitutes one of this study’s limitations, reflects the differing needs of each individual case and is manifested in the variable response to treatment, as demonstrated by the seizure frequency and severity. Additional research is required to evaluate cholinesterase, oxytocin and oxidative stress, and inflammatory markers in larger groups of epileptic dogs. Homogeneity is quite difficult to obtain in naturally occurring animal studies since each individual requires specific antiepileptic medication and seizure frequency is unique and unpredictable for every case.

## 5. Conclusions

The current study assessed oxidative stress (PON1, CUPRAC and FRAP) and inflammatory (CRP) markers alongside cholinesterase and oxytocin in serum and CSF samples of dogs diagnosed with different types of epilepsy. Structural epilepsy may alter Paraoxonase 1 (PON1), and cholinesterase levels in CSF samples. Serum was not as optimal biological material as CSF in the investigation of oxidative stress and inflammatory markers in patients with epilepsy, as indicated by the results of this study.

## Figures and Tables

**Table 1:** Descriptive statistics of the serum oxidative stress parameters, cholinesterase and C-reactive protein (CRP).

Parameters	Paraoxonase 1 (PON1)				Cupric Reducing Antioxidant Capacity (CUPRAC)				Cholinesterase				C-Reactive Protein (CRP)			
**Groups**	**A**	**B**	**C**	**D**	**A**	**B**	**C**	**D**	**A**	**B**	**C**	**D**	**A**	**B**	**C**	**D**
Valid	8	15	11	17	8	15	11	17	8	15	11	17	8	15	11	17
Missing	0	0	0	0	0	0	0	0	0	0	0	0	0	0	0	0
Median	3.220	3.410	3.500	3.470	0.167	0.170	0.159	0.151	4.100	4.000	3.500	4.100	5.550	4.100	3.400	3.800
Mean	3.231	3.557	3.586	3.400	0.171	0.179	0.163	0.164	4.125	3.827	3.436	4.306	10.338	11.860	3.464	11.629
Std. Deviation	0.583	0.937	0.337	0.867	0.027	0.037	0.010	0.047	0.880	1.065	0.757	1.682	7.773	23.722	0.904	16.521
95% CI Std. Dev. Upper	1.186	1.478	0.592	1.320	0.056	0.059	0.018	0.071	1.790	1.680	1.328	2.561	15.820	37.412	1.586	25.143
95% CI Std. Dev. Lower	0.385	0.686	0.236	0.646	0.018	0.027	0.007	0.035	0.582	0.780	0.529	1.253	5.139	17.368	0.631	12.304
Skewness	−0.513	0.339	0.267	−0.013	0.160	1.395	0.473	0.674	0.740	0.094	0.163	2.290	0.668	3.612	0.039	2.678
Std. Error of Skewness	0.752	0.580	0.661	0.550	0.752	0.580	0.661	0.550	0.752	0.580	0.661	0.550	0.752	0.580	0.661	0.550
Kurtosis	−0.558	−0.272	−1.479	−0.059	−2.208	3.976	−1.230	−0.271	1.017	0.407	−0.847	7.039	−1.941	13.478	0.288	7.838
Std. Error of Kurtosis	1.481	1.121	1.279	1.063	1.481	1.121	1.279	1.063	1.481	1.121	1.279	1.063	1.481	1.121	1.279	1.063
Shapiro–Wilk	0.935	0.958	0.924	0.979	0.864	0.868	0.918	0.925	0.949	0.971	0.967	0.778	0.773	0.438	0.979	0.613
*p*-value of Shapiro–Wilk	0.561	0.657	0.351	0.950	0.131	0.032	0.303	0.181	0.706	0.872	0.860	0.001	0.015	1.150 × 10^−6^	0.961	1.386 × 10^−5^
Minimum	2.240	1.890	3.170	1.780	0.140	0.120	0.150	0.088	3.000	1.900	2.300	2.600	3.100	1.500	1.800	2.300
Maximum	3.890	5.240	4.130	5.190	0.207	0.283	0.180	0.253	5.800	6.100	4.700	9.800	21.700	95.800	5.100	66.600

**Table 2:** Post hoc comparisons for oxidative stress markers, cholinesterase and CRP in serum samples.

Group Comparisons		Mean Difference	SE	df	t	p_tukey_
**PON1**						
A	B	−0.326	0.337	47	−0.968	0.768
	C	−0.355	0.358	47	−0.993	0.754
	D	−0.169	0.330	47	−0.511	0.956
B	C	−0.029	0.306	47	−0.095	1.000
	D	0.157	0.273	47	0.577	0.938
C	D	0.186	0.298	47	0.626	0.923
**CUPRAC**						
A	B	−0.008	0.016	47	−0.496	0.960
	C	0.008	0.017	47	0.492	0.960
	D	0.008	0.015	47	0.489	0.961
B	C	0.016	0.014	47	1.123	0.677
	D	0.015	0.013	47	1.205	0.627
C	D	−6.791 × 10^−4^	0.014	47	−0.049	1.000
**Cholinesterase**						
A	B	0.298	0.543	47	0.549	0.946
	C	0.689	0.576	47	1.195	0.633
	D	−0.181	0.532	47	−0.340	0.986
B	C	0.390	0.492	47	0.793	0.857
	D	−0.479	0.439	47	−1.091	0.697
C	D	−0.870	0.480	47	−1.812	0.281

*Note.* *p*-value and confidence intervals of comparing a family of estimates (confidence intervals corrected using Tukey’s method) for PON1; *Note2*. *p*-value adjusted for comparing a family of four estimates for CUPRAC and cholinesterase.

**Table 3:** Kruskal–Wallis test for CRP in serum samples.

		CRP
	Factor	group
	Statistic	6.648
	dF	3
	P	0.084
	Rank ε^2^	0.133
95% CI for Rank ε^2^	Lower	0.059
	Upper	0.305
	Rank η^2^	0.078
95% CI for Rank η^2^	Lower	0.016
	Upper	0.296

**Table 4:** Dunn’s post hoc comparisons for oxidative stress markers, cholinesterase and CRP in serum samples.

Comparisons	z	W_i_	W_j_	r_rb_	p	p_bonf_	p_holm_
CRP							
A—B	−0.809	20.938	26.200	0.167	0.419	1.000	1.000
A—C	−1.299	20.938	29.909	0.409	0.194	1.000	1.000
A—D	−0.744	20.938	25.676	0.184	0.457	1.000	1.000
B—C	−0.629	26.200	29.909	0.152	0.530	1.000	1.000
B—D	0.099	26.200	25.676	0.043	0.921	1.000	1.000
C—D	0.736	29.909	25.676	0.134	0.462	1.000	1.000

*Note.* Rank-biserial correlation based on individual Mann–Whitney tests.

**Table 5:** Descriptive statistics of CSF oxidative stress and inflammatory markers, cholinesterase and oxytocin.

Parameters	PON1				FRAP				Cholinesterase				CUPRAC				Oxytocin			
**Groups**	**A**	**B**	**C**	**D**	**A**	**B**	**C**	**D**	**A**	**B**	**C**	**D**	**A**	**B**	**C**	**D**	**A**	**B**	**C**	**D**
Valid	5	4	5	7	5	6	7	8	5	4	6	7	5	6	7	8	5	6	7	8
Missing	0	2	2	1	0	0	0	0	0	2	1	1	0	0	0	0	0	0	0	0
Median	34.100	34.800	31.000	51.000	0.111	0.167	0.112	0.206	58.000	65.600	77.550	152.700	0.050	0.074	0.078	0.163	912.920	615.285	561.190	1161.220
Mean	34.920	34.450	31.860	345.014	0.121	0.218	0.175	0.263	61.280	72.150	79.783	368.671	0.058	0.092	0.093	0.197	902.374	698.715	688.223	2746.130
Std. Deviation	5.186	3.580	12.054	551.093	0.072	0.171	0.133	0.208	13.030	20.756	37.847	454.231	0.025	0.064	0.052	0.167	151.306	358.166	343.063	3223.943
Skewness	0.931	−0.549	1.502	2.238	1.705	0.492	1.704	1.008	0.131	1.431	0.274	2.038	1.720	0.467	1.261	1.760	0.023	2.142	0.017	1.521
Std. Error of Skewness	0.913	1.014	0.913	0.794	0.913	0.845	0.794	0.752	0.913	1.014	0.845	0.794	0.913	0.845	0.794	0.752	0.913	0.845	0.794	0.752
Kurtosis	0.139	0.952	2.565	5.186	3.215	−2.187	3.133	0.387	−0.393	1.739	−1.070	4.165	3.235	−1.954	0.740	3.379	−2.550	4.890	−0.364	0.726
Std. Error of Kurtosis	2.000	2.619	2.000	1.587	2.000	1.741	1.587	1.481	2.000	2.619	1.741	1.587	2.000	1.741	1.587	1.481	2.000	1.741	1.587	1.481
Shapiro–Wilk	0.902	0.982	0.851	0.658	0.816	0.851	0.826	0.866	0.978	0.867	0.961	0.709	0.831	0.892	0.846	0.805	0.902	0.700	0.964	0.690
*p*-value of Shapiro–Wilk	0.424	0.911	0.198	0.001	0.109	0.162	0.073	0.139	0.924	0.286	0.826	0.005	0.141	0.331	0.112	0.032	0.422	0.006	0.851	0.002
Minimum	30.300	29.800	21.200	34.600	0.062	0.052	0.050	0.072	44.400	55.800	34.000	85.800	0.037	0.022	0.050	0.059	743.270	454.080	164.890	619.650
Maximum	42.800	38.400	51.900	1542.700	0.243	0.436	0.445	0.660	78.600	101.600	133.800	1329.400	0.100	0.175	0.189	0.562	1081.760	1409.120	1195.170	8894.840

**Table 6:** Post hoc comparisons for oxidative stress markers (FRAP and CUPRAC) in CSF samples.

Group Comparisons		Mean Difference	SE	df	t	p_tukey_
**FRAP**						
A	B	−0.096	0.098	22	−0.986	0.759
	C	−0.054	0.095	22	−0.566	0.941
	D	−0.141	0.092	22	−1.534	0.435
B	C	0.043	0.090	22	0.478	0.963
	D	−0.045	0.087	22	−0.514	0.955
C	D	−0.088	0.084	22	−1.050	0.723
**CUPRAC**						
A	B	−0.034	0.062	22	−0.542	0.948
	C	−0.035	0.060	22	−0.582	0.936
	D	−0.139	0.059	22	−2.367	0.113
B	C	−0.001	0.057	22	−0.023	1.000
	D	−0.105	0.056	22	−1.891	0.260
C	D	−0.104	0.053	22	−1.949	0.237

*Note.* *p*-value adjusted for comparing a family of four estimates.

**Table 7:** Kruskal–Wallis test for PON1, cholinesterase and oxytocin in CSF samples.

		PON1	Cholinesterase	Oxytocin
	Factor	group	group	group
	Statistic	8.489	10.763	8.013
	dF	3	3	3
	P	0.037	0.013	0.046
	Rank ε^2^	0.424	0.513	0.321
95% CI for Rank ε^2^	Lower	0.179	0.379	0.113
	Upper	0.824	0.797	0.645
	Rank η^2^	0.323	0.431	0.228
95% CI for Rank η^2^	Lower	0.108	0.254	0.000
	Upper	0.706	0.813	0.665

**Table 8:** Dunn’s post hoc comparisons forPON1, cholinesterase and oxytocin in CSF samples.

Comparisons	z	W_i_	W_j_	r_rb_	p	p_bonf_	p_holm_
**PON1**							
A—B	−0.006	9.100	9.125	0.050	0.995	1.000	1.000
A—C	0.586	9.100	6.800	0.280	0.558	1.000	1.000
A—D	−2.018	9.100	16.429	0.771	0.044	0.262	0.218
B—C	0.559	9.125	6.800	0.400	0.576	1.000	1.000
B—D	−1.879	9.125	16.429	0.786	0.060	0.362	0.241
C—D	−2.651	6.800	16.429	0.771	0.008	0.048	0.048
**Cholinesterase**							
A—B	−0.539	6.400	8.750	0.300	0.590	1.000	1.000
A—C	−0.958	6.400	10.167	0.333	0.338	1.000	1.000
A—D	−3.013	6.400	17.857	1.000	0.003	0.016	0.016
B—C	−0.338	8.750	10.167	0.167	0.735	1.000	1.000
B—D	−2.238	8.750	17.857	0.857	0.025	0.151	0.126
C—D	−2.129	10.167	17.857	0.714	0.033	0.200	0.133
**Oxytocin**							
A—B	1.468	15.800	9.000	0.667	0.142	0.852	0.568
A—C	1.359	15.800	9.714	0.429	0.174	1.000	0.568
A—D	−0.677	15.800	18.750	0.300	0.499	1.000	0.997
B—C	−0.168	9.000	9.714	0.000	0.867	1.000	0.997
B—D	−2.360	9.000	18.750	0.708	0.018	0.110	0.110
C—D	−2.283	9.714	18.750	0.679	0.022	0.135	0.112

*Note.* Rank-biserial correlation based on individual Mann–Whitney tests.

## Data Availability

Additional data are unavailable due to privacy and ethical restrictions.

## Supplementary Materials

The following supporting information can be downloaded at: https://www.mdpi.com/article/10.3390/antiox15030282/s1, Table S1: Epidemiological data of the four groups of dogs.

## Abbreviations

The following abbreviations are used in this manuscript:

AChE	Acetylcholine
AEM	Antiepileptic Medication
ANOVA	Analysis of Variance
BBB	Blood–Brain Barrier
Br	Bromide
CBC	Complete Blood Count
CNS	Central Nervous System
CRP	C-Reactive Protein
CSF	Cerebrospinal Fluid
CT	Computed Tomography
CVs	Coefficients of Variations
CUPRAC	Cupric Reducing Antioxidant Capacity
EEG	Electroencephalography
FRAP	Ferric Reducing Antioxidant Power
HDL	High-Density Lipoprotein
ILAE	International League Against Epilepsy
LD	Detection Limit
LEV	Levetiracetam
LLQ	Lower Limit of Quantification
MRI	Magnetic Resonance Imaging
PB	Phenobarbital
PON1	Paraoxonase 1
RNS	Reactive Nitrogen Species
ROS	Reactive Oxygen Species
