# Supplementary material for: Assessment of Oxidative Stress-Related Markers and Inflammatory Proteins in Serum and CSF Samples of Dogs with Different Types of Epilepsy
Source: Antioxidants (Basel). 2026 Feb 25;15(3):282. doi: 10.3390/antiox15030282 (PMC13024043; doi:10.3390/antiox15030282)
Supplement: Supplementary file 1 [file antioxidants-15-00282-s001.zip › antioxidants-4072116-supplementary.pdf]

Table S1. Epidemiological data of the four groups of dogs.

	Group A	Group B	Group C	Group D
**AGE (months)**				
Min value	12	12	9	42
Max value	84	84	78	168
Median Value	24	48	42	100
**BODY WEIGHT (Kg)**				
Min value	21.6	2.3	7	4.2
Max value	45	51.5	48	60
Median value	26.5	11.6	26.5	17.8
**GENDER**				
	Number of dogs (%) Total:8	Number of dogs (%) Total: 15	Number of dogs (%) Total: 11	Number of dogs (%) Total: 17
Male	4 (50%)	9 (60%)	9 (81.8%)	11 (64.7%)
Female	4 (50%)	6 (40%)	2 (18.2%)	6 (35.3%)
**BREED**				
	Number of dogs (%) Total:8	Number of dogs (%) Total:15	Number of dogs (%) Total: 11	Number of dogs (%) Total: 17
Mixed breed	8 (100%)	9 (60%)	4 (36.4%)	10 (58.8%)
Purebred dogs	0	6 (40%)	7 (63.6%)	7 (41.2%)
**SEIZURE ONSET AGE (months)**				
Min value	0	7	3	24
Max value	0	66	78	168
Median value	0	30	30	96
**TYPE OF EPILEPTIC SEIZURE**				
	Number of dogs (%) Total:8	Number of dogs (%) Total:15	Number of dogs (%) Total: 11	Number of dogs (%) Total: 17
Focal	0	1 (6.7%)	0	0
Generalized	0	9 (60%)	10 (90.9%)	17 (100%)
Both focal and generalized	0	5 (33.3%)	1 (9.1%)	0
**EPILEPTIC SEIZURE FREQUENCY**				
	Number of dogs (%) Total:8	Number of dogs (%) Total:15	Number of dogs (%) Total: 11	Number of dogs (%) Total: 17
Single seizure	0	1 (13.3%)	5 (45.5%)	8 (47%)
Cluster seizures	0	4 (26.7%)	2 (18.2%)	5 (29.4%)
Mixed (single + cluster seizure/ status epilepticus)	0	9 (60%)	4 (36.3%)	4 (23.6%)
**ANTIEPILEPTIC THERAPY ON ADMISSION**				
	Number of dogs (%) Total:8	Number of dogs (%) Total:15	Number of dogs (%) Total: 11	Number of dogs (%) Total: 17
Monotherapy	0	3 (20%)	0	13 (76.5%)
Polytherapy	0	12 (80%)	0	1 (5.9%)
No therapy	8 (100%)	0	11 (100%)	3 (17.6%)
**ANTIEPILEPTIC DRUGS (AED) ON ADMISSION**				
	Number of dogs Total:8	Number of dogs Total:15	Number of dogs (%) Total: 11	Number of dogs (%) Total: 17
Phenobarbital	0	3	0	7

monotherapy				
Levetiracetam monotherapy	0	0	0	6
Phenobarbital combined with other AEDs	0	10	0	1
Potassium bromide (in polytherapy)	0	7	0	1
Levetiracetam (in polytherapy)	0	11	0	0
Imepitoin (in polytherapy)	0	6	0	0
Pregabalin (in polytherapy)	0	1	0	0
Gabapentin (in polytherapy)	0	2	0	0
Zonisamide (in polytherapy)	0	1	0	0
**DIAGNOSTIC IMAGING**				
	Number of dogs (%) Total:8	Number of dogs (%) Total:15	Number of dogs (%) Total: 11	Number of dogs (%) Total: 17
CT	0	12 (80%)	11 (100%)	7 (41.1%)
CT+MRI	0	3 (20%)	0	10 (58.8%)
**DIAGNOSIS**				
	Number of dogs (%) Total:8	Number of dogs (%) Total:15	Number of dogs (%) Total: 11	Number of dogs (%) Total: 17
Healthy controls	8	0	0	0
Idiopathic epilepsy	0	15	11	0
Structural epilepsy: head trauma	0	0	0	1 (5.9%)
Structural epilepsy: ischemic encephalopathy	0	0	0	3 (17.6%)
Structural epilepsy: neoplasia	0	0	0	7 (41.1%)
Structural epilepsy: hydrocephalus	0	0	0	2 (11.8%)
Cryptogenic epilepsy	0	0	0	4 (23.5%)
**FOLLOW UP**				
	Number of dogs (%) Total:8	Number of dogs (%) Total:15	Number of dogs (%) Total: 11	Number of dogs (%) Total: 17
Stability	No data	4 (26.6%)	No data	3 (17.6%)
Improvement	No data	4 (26.6%)	No data	3 (17.6%)
Deterioration	No data	6 (40%)	No data	8 (47.2%)

Missing data	No data	1 (6.7%)	No data	3(17.6%)
